# Concomitant diagnosis of hypersensitivity pneumonitis and localized epithelioid mesothelioma: a rare case report

**DOI:** 10.31744/einstein_journal/2025RC1296

**Published:** 2025-10-30

**Authors:** Simone de Leon Martini, Thiago Krieger Bento da Silva, Bianca Canela Furian, Ana Carolina Storch Klein, Júlia Gabriela Storch Klein, Camila Samrsla Möller, Dâmaris Manfro Pinto Garcia, Guilherme Bohm Ferreira

**Affiliations:** 1 Universidade Feevale Novo Hamburgo RS Brazil Universidade Feevale, Novo Hamburgo, RS, Brazil.; 2 Pontifícia Universidade Católica do Rio Grande do Sul Hospital São Lucas Porto Alegre RS Brazil Hospital São Lucas, Pontifícia Universidade Católica do Rio Grande do Sul, Porto Alegre, RS, Brazil.; 3 Santa Casa de Porto Alegre Porto Alegre RS Brazil Santa Casa de Porto Alegre, Porto Alegre, RS, Brazil.

**Keywords:** Mesothelioma, Rare diseases, Alveolitis, extrinsic allergic, Lung diseases

## Abstract

Hypersensitivity pneumonitis is an inflammatory lung disease caused by antigen inhalation after sensitization. Here, we report a rare case of the simultaneous diagnosis of hypersensitivity pneumonitis and epithelioid mesothelioma, a subtype of malignant mesothelioma. A 71-year-old man presented with an occasional dry cough and exertional dyspnea that had persisted for about 1 year. He reported walking daily in a park with a considerable number of birds during same period. Functional evaluation revealed a mild restrictive ventilatory disorder. High-resolution computed tomography showed nonspecific peripheral interstitial lung involvement along with signs of bronchopathy and diffuse bronchiolopath without honeycombing or bronchiectasis. Owing to the patient's clinical history and the presence of a restrictive ventilatory disorder, a biopsy was performed for diagnostic clarification. Pathological examination revealed hypersensitive pneumonitis associated with epithelioid mesothelioma. Surgical resection of the mesothelioma and avoidance of occupational exposure resulted in complete regression of symptoms and improvement in radiological findings. A report on this rare concomitant diagnosis of the hypersensitivity pneumonitis and epithelioid mesothelioma is essential for advancements in medicine.

## INTRODUCTION

Hypersensitivity pneumonitis (HP), also called extrinsic allergic alveolitis, is a disease that involves inhalation and sensitization to organic or inorganic agents, and is usually caused by occupational factors.^(1)^ Its clinical presentation varies and depends on the amount of exposure, duration, and host factors. HP treatment involves removing the exposure and using corticosteroids when necessary.^(2)^

Mesothelioma arises from cells on the serous surface lining of the pleural, peritoneal, and pericardial cavities. Pleural mesothelioma is the most common form (90%) and is strongly associated with asbestos exposure.^(3)^ This cancer exhibits a range of morphological differentiations and is divided into epithelioid, biphasic, and sarcomatoid types.^(4)^ Epithelioid mesothelioma (EM), according to the 2015 classification of the World Health Organization, it is the most common and has the best prognosis.^(5)^ Immunohistochemical studies of EMs typically show a positive reaction to calretinin, cytokeratin 5/6, HBME-1, and mesothelin, in addition to new markers identified in recent research.^(6)^

Although rare, malignant mesothelioma has increased since the second half of the 20^th^ century mainly due to asbestos exposure. It affects men more frequently (5:1 ratio), is prevalent after 65 years, and in Brazil shows rising mortality, with 80.7% of deaths in those over 50, though underdiagnosis remains common.^(5)^ Genomic studies have identified recurrent mutations, notably BAP1. Testa et al. found germline BAP1 mutations in 7.7% of spontaneous mesotheliomas, while Ohar et al. reported BAP1 mutations in 9 of 150 familial cases, 8 of which were epithelioid.^(7,8)^ Diagnostic accuracy also relies on immunohistochemistry. The 2017 guidelines highlight CK5/6 (75%-100%), calretinin (most cases), WT1 (70%-95%), and D2-40 (90%-100%) as key markers.^(9)^

Therefore, we report a rare coexistence of HP and EM, revealing the challenges in diagnosis and treatment.

## CASE REPORT

**Figure 1:**
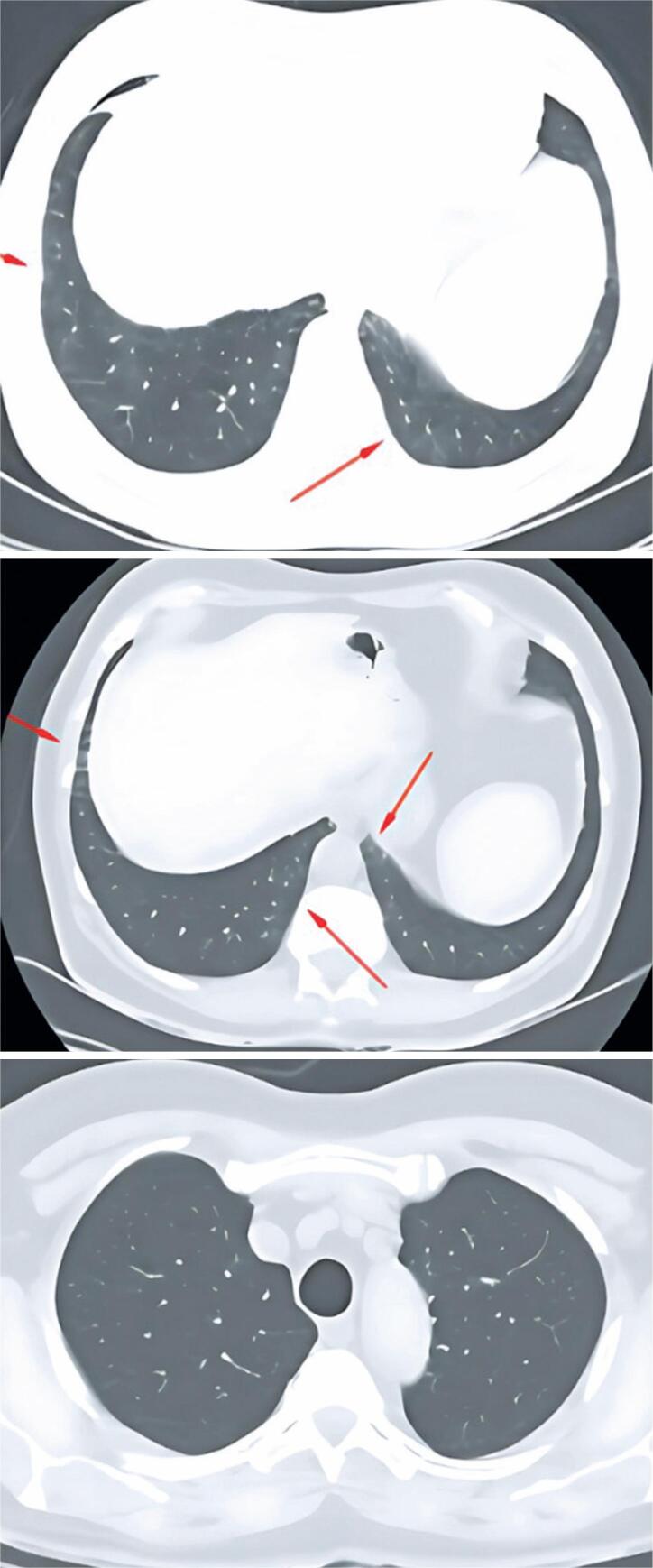
Chest CT in axial plane. Arrows indicate subtle ground-glass and reticular opacities

**Figure 2:**
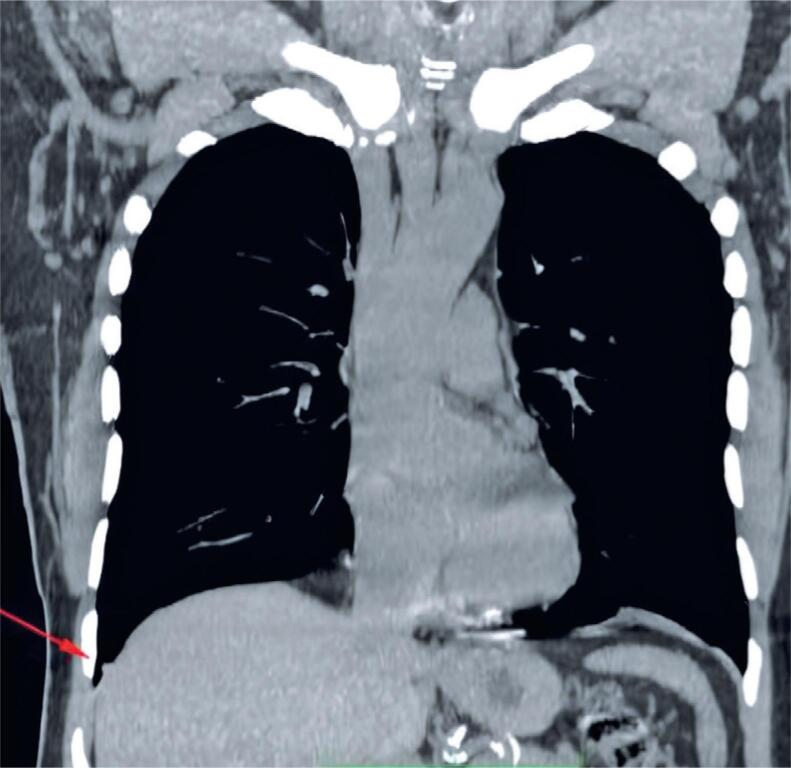
Chest CT in coronal plane. The arrow indicates mild thickening of the costodiaphragmatic pleura

**Figure 3:**
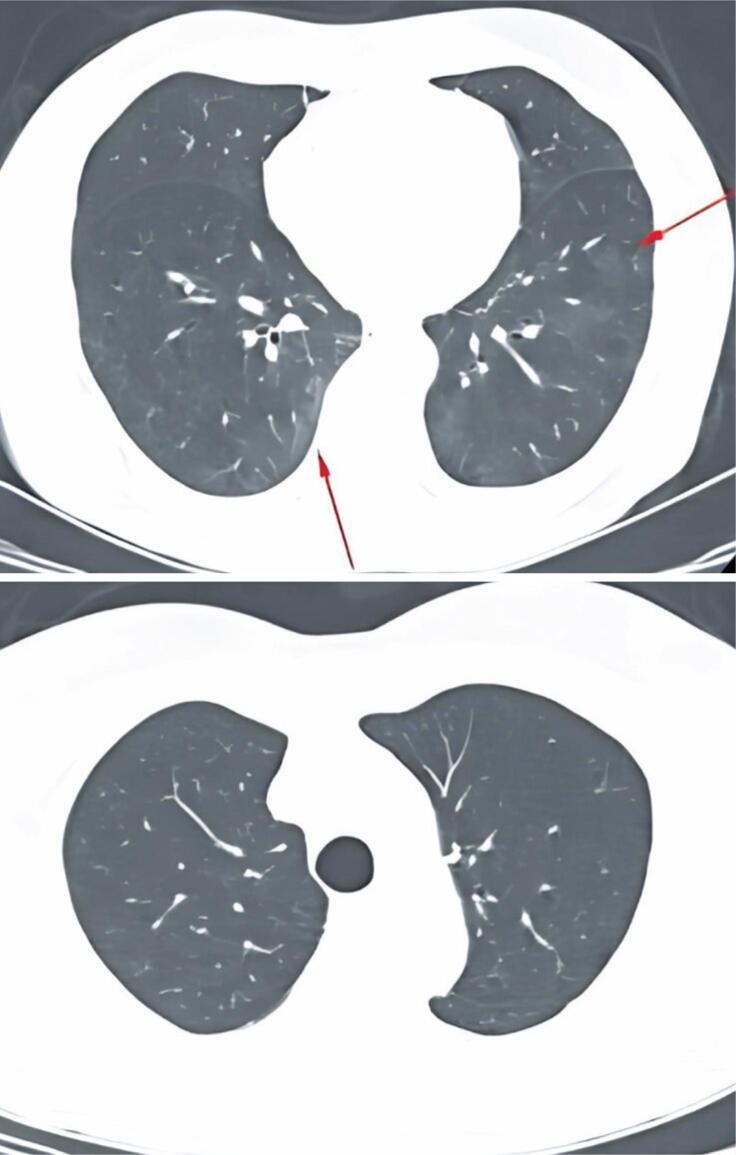
Chest CT in axial plane. Lower lobes show an increase in ground-glass lesions, now with more peribronchovascular central involvement. Apices remain lesion-free

A 71-year-old Caucasian male from Taquara, RS, presented with occasional dry cough and exertional dyspnea that had persisted for about 1 year. He had a maternal family history of metastatic bowel cancer. He denied any history of active or passive smoking and reported no family history of bronchial asthma. The patient was advised to visit a pulmonologist in January 2018 because of a persistent dry cough after treatment for rhinosinusitis. The cough occurred sporadically and worsened with temperature changes. In addition, he reported progressive dyspnea on exertion that had started 1 year earlier. In 2018, spirometry revealed a mild restrictive ventilatory disorder that required further investigation. Based on the patient's clinical history, HP was suspected owing to his habit of taking daily walks for an average of 50 min in a park with a moderate number of birds, which started 1 year earlier. The first chest computed tomography (CT) performed in December 2019 showed mild nonspecific inflammatory interstitial lung disease and signs of bronchopathy and diffuse bronchiolopathy (Figure 1). The scan also showed a small diaphragmatic pleural thickening on the right side (Figure 2). A follow-up CT conducted in May 2020 revealed increased interstitial pulmonary infiltration with ground-glass attenuation, predominantly in the lower lobes, as well as multifocal areas of air trapping (Figure 3). Subsequently, a surgical biopsy of the right upper lobe and right lower lobe (RLL) of the lungs was performed in November 2020 was performed in November 2020.

**Figure 4:**
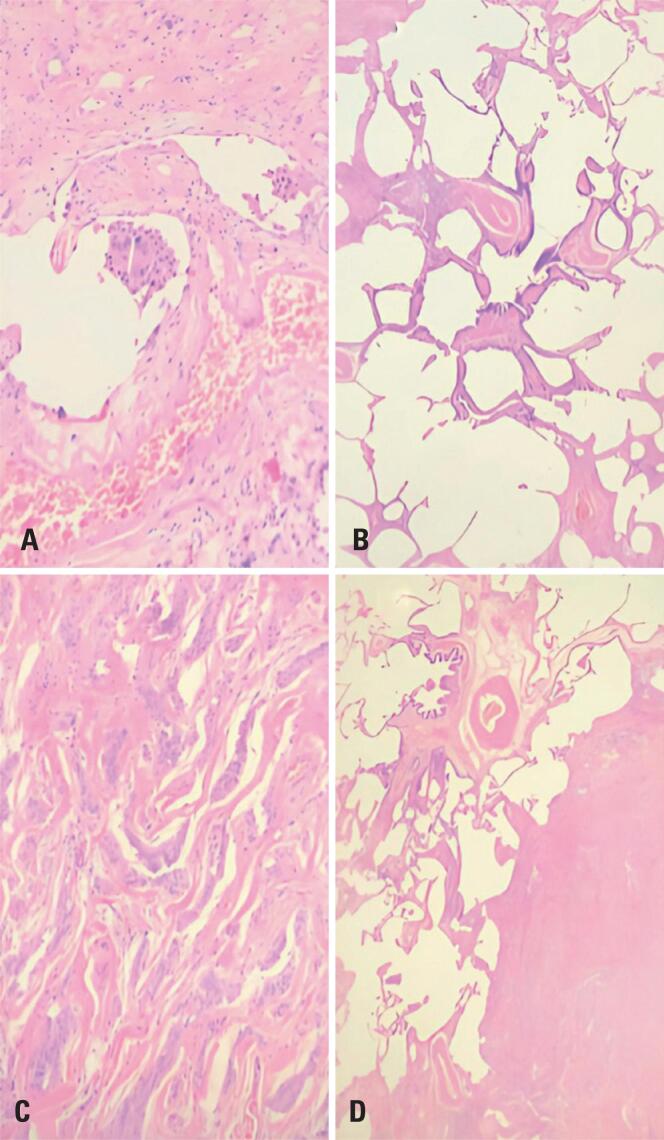
A) Peribronchial granuloma; B) Peribronchial bronchial metaplasia; C) Mesothelioma; D) Mesothelioma and lung parenchyma with peribronchial metaplasia

**Figure 5:**
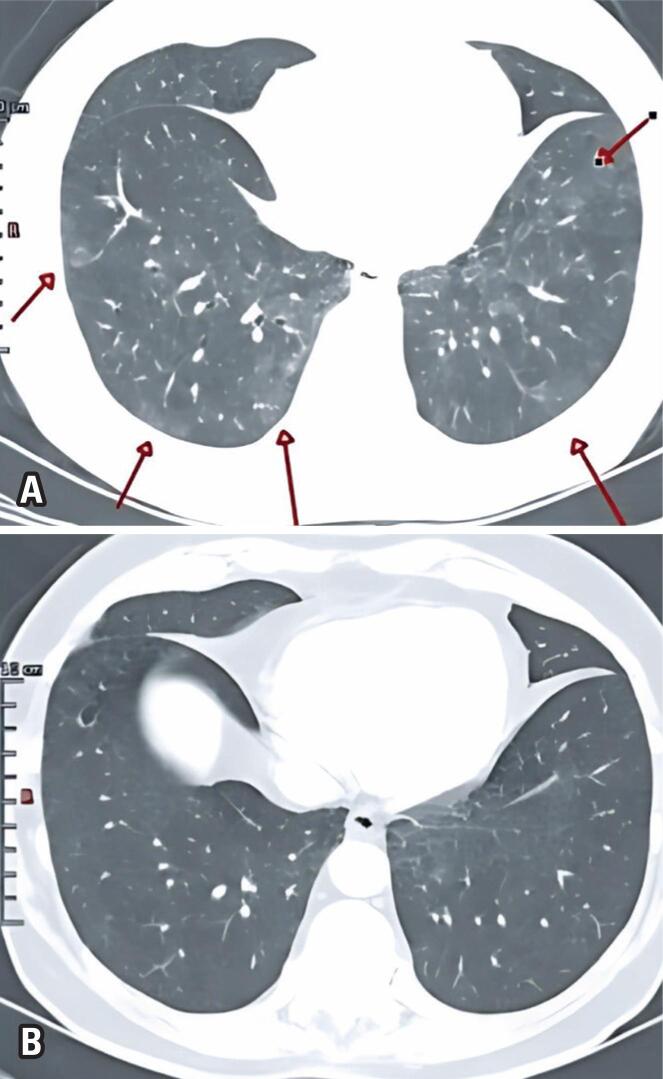
A) Tomographic image in May 2020 | The arrows show subtle ground-glass and reticular opacities; B) Tomographic image in January 2021

The biopsy revealed a 7.32mm subpleural EM located in the RLL, which was surgically removed, along with areas of fibrosis and bronchiolocentric inflammation characteristic of HP (Figure 4). Immunohistochemical analysis of the samples revealed positive markers for CK7, CK5/6, WT1, calretinin, BAP-1, and D2-40. Because of the rarity and limited knowledge regarding the concomitance of these diseases, the diagnosis was reviewed at another specialized diagnostic center, which confirmed the results. The patient was advised to discontinue his daily walks in the park to minimize exposure to potential pathogens or antigens, resulting in an improvement in his clinical condition. A chest CT scan performed in January 2021 showed an almost complete resolution of the diffuse interstitial pulmonary infiltrates (Figure 5).

This case is of utmost importance because no previous reports exist regarding the coexistence of HP and EM, which is a rare and challenging diagnosis for healthcare professionals.

This study was approved by the Research Ethics Committee of *Universidade Feevale* (CAAE: 71115723.4.0000.5348, # 6.710.329). Written informed consent was obtained from the patient for publication of this case report and accompanying images.

## DISCUSSION

This case describes a 71-year-old man with clinical and radiological features of HP in whom biopsy unexpectedly revealed epithelioid mesothelioma. Immunohistochemical analysis confirmed the diagnosis, with positive markers including CK7, CK5/6, WT1, calretinin, BAP-1, and D2-40.

The rarity of this coexistence makes the case relevant to medical literature. It illustrates how overlapping clinical and radiological findings can obscure the underlying diagnosis, and how careful integration of pathology, imaging, and exposure history is essential. Clinical improvement following exposure avoidance further reinforces the importance of environmental control in HP management, even when a second rare diagnosis is present.

Malignant mesothelioma is usually unilateral (95%) and predominantly located in the right hemithorax (60%). EMs exhibit architectural, cytological, and stromal characteristics that overlap with those of other neoplasms. The unavailability of an efficient method for early detection further complicates its diagnosis. Generally, the prognosis is poor, as most patients present with unresectable disease at diagnosis or are considered inoperable owing to age, poor performance status, or comorbidities.^(5)^

In contrast, in our patient, EM was incidentally identified at an early stage and surgically removed, allowing a more favorable outlook despite risk factors such as advanced age and a family history of metastatic cancer. The absence of asbestos exposure also points to the potential role of genetic alterations, such as BAP-1 mutations, which have been increasingly recognized in recent studies.

HP, also known as extrinsic allergic alveolitis, is an immune-mediated interstitial inflammatory syndrome triggered by exposure to and inhalation of numerous allergens, most commonly fungi, thermophilic bacteria, or bird feathers.^(10)^ Depending on the type of exposure, the disease is given a specific name, such as bird fancier's lung, due to the inhalation of serum proteins and contaminants from bird feathers and droppings.^(11)^ In Brazil, HP is estimated to account for 3%-13% of interstitial lung diseases.^(10)^ In up to 40% of biopsy-confirmed cases, the agent may not be identified.^(12)^

Clinical suspicion in this patient arose from persistent dry cough, despite treatment for rhinosinusitis. To support the diagnostic hypothesis, data collected during anamnesis regarding the patient's physical activity in a location with abundant bird exposure, along with the presence of restrictive ventilatory disorder (a typical functional aspect of chronic HP) and complaints of dyspnea on minor exertion, led to the consideration of hypersensitivity disorder.^(13)^ These observations highlight the importance of the correlation between clinical history and complementary examinations for diagnosis.

Certain tomographic and histopathological findings can aid in diagnosing HP and determining disease stage. Ground-glass opacities, centrilobular ground-glass micronodules, air trapping, and fibrosis are characteristic signs of chronic diseases. Histopathologically, cellular bronchiolitis, chronic interstitial inflammatory infiltrates, granulomas, giant cells, and fibrosis are considered signs of advanced disease.^(12)^

In this case report, the patient's CT scan in 2019 showed mild interstitial pulmonary infiltration with ground-glass attenuation, predominantly in the cortical regions of the lower lobes, along with multifocal air trapping on expiration images, indicating small airway involvement. These findings were interpreted as signs of mild, non-specific inflammatory interstitial lung disease. Compared to the first scan, the second CT scan performed in 2020 revealed an increase in the diffuse areas of interstitial pulmonary infiltration with ground-glass attenuation, predominantly in the lower lobes, related to active inflammatory processes. A surgical biopsy was performed to confirm the diagnosis of chronic HP through histopathological examination.

The prognosis and progression of HP depend on the concentration of inhaled antigens and the duration of patient exposure to the agent.^(14)^ Treatment involves avoiding the causative agent and identifying it, particularly if the disease is progressive and fibrosing.^(11)^ In cases of non-fibrotic HP, retrospective studies have shown improved pulmonary function and clinical outcomes with corticosteroid use. Additionally, the use of antifibrotic agents such as nintedanib has shown efficacy in reducing functional decline in patients with HP and progressive fibrosing diseases.^(15)^ In our case, the patient experienced continuous symptom regression after ceasing to walk in the park and did not require oral corticosteroids as his dyspnea complaints were mild.

## CONCLUSION

This case highlights the importance of thorough anamnesis and complete clinical history for diagnosing mesothelioma and suspected hypersensitivity pneumonitis. The concomitant occurrence of these pathologies represents an unexplored field in the study of lung diseases, requiring a better understanding of their coexistence and the development of more effective treatment strategies to improve patient prognosis.
